# Association Between Household Livestock Ownership and Stunting in Children Younger Than 5 Years in Central Rural India: Protocol for a Case-Control Study

**DOI:** 10.2196/66576

**Published:** 2025-09-11

**Authors:** Pratik Prashant Tawde, Zahiruddin Quazi, Abhay Gaidhane, Sonali Choudhari

**Affiliations:** 1 Department of Community Medicine Jawaharlal Nehru Medical College Datta Meghe Institution of Higher Education and Research Wardha India

**Keywords:** stunting, wasting, livestock, tropical livestock unit, TLU, India, animal-source food, study protocol, case-control study

## Abstract

**Background:**

In resource-constrained environments where malnutrition in children is common, people coexist closely with livestock. Undernutrition, which can result in stunting, wasting, and being underweight, is still a major public health issue for children in India. Livestock serves as a direct source of protein through animal-source foods such as meat, milk, and eggs, which significantly reduce undernutrition in children younger than 5 years. The association between livestock ownership and stunting in children has not been explored well in Maharashtra, India.

**Objective:**

This study evaluates the relationship between stunting among children younger than 5 years in central India and livestock ownership by using the Tropical Livestock Unit (TLU) score.

**Methods:**

A community-based, case-control study will be conducted for 12 months among 614 children aged 6-59 months in a few rural blocks of Wardha district, Maharashtra. A predesigned semistructured questionnaire will collect data from the informant (either parent). Anthropometric measurements will be plotted on World Health Organization (WHO) standard growth charts for *z* scores for stunting and wasting. TLU will be used to represent the livestock count of all species. The categorical variables (stunted and nonstunted) will be analyzed using the Fisher exact test to find significant associations between different risk factors. All confounding variables will be analyzed using multivariable logistic regression. Associated risks will be identified and tabulated using the odds ratio with confidence intervals.

**Results:**

This study protocol will provide a comprehensive understanding of the associations of livestock ownership with childhood stunting and wasting among children aged 6-59 months in central rural India. This study will be conducted from October 2025 to September 2026. Currently, necessary approvals are obtained, and data collection is planned to begin from November 2025 to May 2026.

**Conclusions:**

Our findings will show the prevalence of the current stunting, wasting, and underweight rates among Indian children younger than 5 years and the association of these rates with livestock ownership by using the TLU index. These results will be instrumental in building new public health policies, integrated programs, and nutritional interventions.

**Trial Registration:**

Clinical Trials Registry – India CTRI/2024/05/066865; https://tinyurl.com/y3zvh5t7

**International Registered Report Identifier (IRRID):**

PRR1-10.2196/66576

## Introduction

Stunting, which refers to low height/length for age, is usually a result of chronic undernutrition, often linked to poor socioeconomic conditions, maternal health, infant care, and children’s nutrition. It restricts children from achieving maximum physical and cognitive capabilities [1]. In 2022, 149 million children younger than 5 years experienced stunted growth, which accounts for 22.3% of all children younger than 5 years globally [2]. More than a million child deaths are estimated annually due to stunting, which is defined as a height-for-age below minus 2 *z* scores of the median height-for-age in the World Health Organization (WHO) child growth standards [3]. Stunting and undernutrition continue to have a high prevalence globally.

In India, although there has been economic growth and significant decreases in child mortality rates, undernutrition and stunting are still prevalent [4]. For children, stunting, wasting, and underweight are crucial markers of their nutritional condition. Although wasting results from acute food scarcity and illness, stunting is due to recurring illnesses and long-term inadequate nutritional intake. Stunting has generally irreversible impacts on delayed motor development and decreased cognitive development. However, wasting is a major indicator of death and needs to be addressed right now. Underweight integrates weight for length/height and linear growth hindrance data [1].

One of the key priorities for the 2030 “Zero Hunger” Sustainable Development Goal is reducing stunting [5]. Stunting and wasting are also highlighted in the Global Hunger Index 2017 report, in which India ranked 100th [6]. The latest National Family Health Survey-5 (NFHS-5) was conducted in India from 2019 to 2021, which revealed that underweight, wasting, and stunting are common in Indian children younger than 5 years. The prevalence of underweight, wasting, and stunting according to NFHS-5 was 35.5%, 19.3%, and 32.1%, respectively [7]. This is undoubtedly lesser than that reported in the NFHS-4 study, which was performed between 2015 and 2016 [8]. The stunting and wasting rate for Maharashtra as per recent NFHS-5 (2019-2021) was 35.2% and 25.6%, respectively. As per NFHS-4 (2019-20) from the district fact sheet of Wardha, Maharashtra, the stunting rate for rural children younger than 5 years was 29.4%, and the wasting rate was 25.8% [9].

Agriculture, with its allied sectors such as animal husbandry and livestock production, is India’s largest source of livelihood [10]. One integrated agricultural component that enhances household income, consumption, productivity, and food security is livestock [11]. Livestock provides protein directly through meat, milk, and eggs, known as animal-source food or indirectly by boosting the household income for food purchases [12]. Livestock ownership can improve the nutritional diversity and the quality of nutrition for households that own them, even though it may also raise the risk of environmental enteropathy, a condition linked to the loss of nutrients in the stomach, in children [13]. The Wardha district in Maharashtra is notable for its livestock and poultry farming, which is essential to the rural economy. According to the 20th livestock census, Wardha district recorded 489,836 animals, of which 344,875 are poultry and cattle units [14]. A few studies conducted in African nations have shown that owning more livestock can greatly boost the consumption of food items derived from animals and enhance nutritional outcomes [15,16]. Nonetheless, few studies [4,17] have been conducted in India to examine the relations between household livestock ownership and the stunting and nutritional status of children younger than 5 years. The tropical livestock unit (TLU), an index used in this study, is created by giving each animal a weight and merging all of the livestock characteristics for the household into a single variable [18]. Additionally, a dearth of studies [19] in Maharashtra examines the association between livestock ownership and stunting in children younger than 5 years. This study seeks to evaluate the relationship between stunting among children younger than 5 years in central rural India and livestock ownership by using the TLU index, considering existing research gaps.

## Methods

### Study Design, Participants, and Setting

This case-control study will be conducted in a community-based setting. It will be performed for 24 months in a few randomly selected villages from 8 rural blocks of Wardha district, located in central Maharashtra, by using a computerized method. Cases and controls will be proportionally allocated based on the number of children younger than 5 years and the stunting prevalence in each block. Permission for the interviews will be obtained from the authority/sarpanch of the respective villages. The list of children younger than 5 years of the selected blocks will be acquired from the respective Anganwadi Center of the village. For each case, 1 control will be recruited through simple random sampling. Informants will be briefed on the study’s process and purpose. Participation will only be allowed after either parent gives written and informed consent. Data will be gathered using a semistructured questionnaire in English/Marathi via interviews. The questionnaire was developed and reviewed by field experts and will be validated through a pilot study (Multimedia Appendix 1). An infantometer/stadiometer will be used by the investigator to measure the length/height (in centimeters), and weighing scales will be used to measure the weight (in kilograms) of each child. The children’s height and weight measurements will be compared to WHO 2006 standards based on age and gender to calculate the height-for-age *z* score and weight-for-height *z* score. Stunting and wasting are defined as falling below –2 SD of the WHO reference median. After plotting the measurements on the WHO growth charts, stunted and nonstunted children will be listed separately. All stunted children will be the cases in this study, and all nonstunted children will be the controls.

### Ethical Considerations

This study received approval on January 31, 2024, from the Institutional Ethics Committee of Datta Meghe Institute of Higher Education and Research (DMIHER(DU)/IEC/2024/137) to ensure adherence to ethical guidelines, respect participant rights, and meet established standards. This study has been registered with the Clinical Trials Registry-India under registration CTRI/2024/05/066865. Participants will be provided with an informed consent form, clearly describing the study’s aim, procedures, risks, benefits, and their right to opt out at any time. Consent for publication of data will be obtained and will be used for all analyses, including secondary analyses. We will confirm that the original consent and institutional review board approval explicitly cover the use of primary and secondary data. We ensure that the data collected will be fully anonymized, with no personally identifiable information retained or linked to any participant. As there are no interventions involved and as only a cross-sectional survey will be conducted in which the data will be collected via an interview and anthropometric measurements, participants will not be provided with any compensation in this study.

### Inclusion and Exclusion Criteria

Cases will be defined as children who are stunted, reside in the study area, are aged 6 months to 5 years, with a mother or father as the caregiver, reside in a household with or without livestock, and are willing to participate and follow the requirements of the study. Controls will be defined as children who are not stunted, residents of the same study area, aged 6 months to 5 years, with a mother or father as the caregiver, who reside in a household with or without livestock, and are ready to participate and follow the requirements of the study. Children will be excluded from the study if they exhibit any obvious congenital defect, have a major medical condition, or need a prolonged hospital stay after birth. Those who have not lived in the Wardha district for at least the past 3 months and those presently involved in another study on animal husbandry will also be excluded.

### Sample Size

The study will involve participants chosen based on specific inclusion and exclusion criteria. It will include all children younger than 5 years from the respective villages. Considering the overall number of children younger than 5 years in the Wardha rural area sample, the total population was 88,224 children [20].

Estimated sample size = n≥(NZ_(1-α⁄2)^2 p(1-p))/(d^2 (N-1)+Z_(1-α⁄2)^2 p(1-p)), where α (a)=.05; estimated proportion of children younger than 5 years who are stunted (height-for-age) *P*=.28 [9]; and estimation error (d) = 5% = 0.05. The minimal sample size calculated for this study will be 614, of which 307 are cases and 307 are controls, considering the ratio of case-control is 1:1.

### Data Collection Tools and Methods

Villages from all 8 blocks of Wardha district will be randomly selected using a computer-based randomization method. A list of all children younger than 5 years will be collected from the Anganwadi Center of the respective villages. The children will be selected from the list according to the inclusion and exclusion criteria. The final list of the selected children of respective villages will be arranged according to the location and proximity of their homes, and the investigator will visit their homes for data collection and anthropometric measurements. Data will be collected using a semistructured questionnaire in English/Marathi. The questions will be asked to the informant (either of the parents). The questionnaire will contain questions related to the sociodemographic profile of the child, mother, and household; personal history of the child; livestock ownership; livestock management and disease; environment; and behavioral determinants such as Water, Sanitation, and Hygiene (WASH) (Multimedia Appendix 1). Following the interview, the child’s height and weight will be measured. The digital weighing scale (Seca), pretested for accuracy, will be used to record weight with minimal clothing. Children up to the age of 2 years will have their length measured while lying on the horizontal measuring scale (Seca infantometer). Children older than 2 years will have their height measured with a stadiometer (Prestige). Height measurement will be conducted up to the nearest tenth of a centimeter. The child will have to stand on the scale barefoot, with heels together and shoulder blades, buttocks, and heels touching the vertical surface. The child’s height will be measured by placing a headpiece on the top of their head while they are looking straight ahead and their arms are hanging naturally at their sides. To ensure anthropometric accuracy, the data collectors will undergo 3-day training and standardization sessions following WHO protocols. Technical errors of measurement will be calculated before the study and will be monitored periodically. There will also be regular supervisory visits, which will ensure consistency and interrater reliability. To reduce recall and selection biases, there will be validation assessments utilizing anthropometric data and health records. Moreover, data triangulation will be utilized by combining interviews, focus groups, and observations from the community.

To calculate the total number of livestock, a standardized unit must be used to represent the livestock counts for all species. A TLU is a standard unit that allows for the comparison of various species of varying sizes by using a common measure. Conversion equivalents of livestock into TLU are mentioned in Table 1 for households with livestock ownership [21].

**Table 1:** Conversion equivalents of livestock into Tropical Livestock Unit [16].

Species (animal type)	Tropical Livestock Unit equivalent
Cattle (bull)	1.0
Cattle (cow/buffalo)	0.8
Cattle (calves)	0.2
Sheep/goats	0.1
Horses	0.8
Donkeys/mules	0.5
Poultry	0.01

### Data Analysis

All the results will be calculated using RStudio software (version 4.3.2; Posit). The complete analysis dataset will consist of all study participants who have no missing data for any of the variables in the dataset. The study participants will be those individuals who meet the criteria for inclusion and exclusion. Baseline characteristics will be utilized to compile all the data, representing demographic variables with mean and standard deviation for continuous data and frequency and percentage for categorical data. The results will be examined for continuous data, which will be described using the minimum, maximum, average, standard deviation, standard error, and 95% CI for parametric data. The normality of the continuous outcome variables will be assessed initially with the Kolmogorov-Smirnov test at a 5% significance level (*P*≤.05). If the data are not rejected, a nonparametric test will be used to determine significance; otherwise, it will be considered normal. The significant difference at the 5% level (*P*≤.05) between stunted and nonstunted comparative groups will be determined using a 2-sided *t* test. Nonnormal data will be summarized using mean, median, and lower and upper quartiles for nonparametric testing and will be assessed for significance using the Mann-Whitney test. Categorical variables will be presented with frequency (n) and percentage value (%). The categorical variables (stunted and nonstunted) will be analyzed by Fisher exact test to find significant associations of different risk factors. All confounding variables will be analyzed using multivariable logistic regression. Associated risks will be investigated and tabulated using the odds ratio with a confidence interval. To understand the effects of multiple factors, confounding variables will be recognized and adjusted to understand the multiple factors that can influence the outcome (stunting). For the confounding effect, multivariable methods will be employed to estimate the association between exposure (livestock) and outcome (stunting), or the impact of one or more confounding factors such as WASH; wealth index of the household; episodes of illness; dietary habits; knowledge, attitude, and practices regarding livestock; and livestock diseases will be considered. Essentially, multivariable logistic regression will allow assessing the independent effect of each of the exposures.

## Results

This study is expected to provide insights into whether there is an association between household livestock ownership (measured by TLU index) and the prevalence of childhood stunting and wasting in central rural India. This study will be conducted from October 2025 to September 2026. Currently, necessary approvals are obtained, and data collection is planned to begin from November 2025 to May 2026.

## Discussion

### Overview

The research aim of this study is to determine the association between stunting among children younger than 5 years and household livestock ownership in rural areas of central India, and if a one-health approach can contribute to and address this association.

According to Hossain and Khan [22], owning cattle in Bangladesh is substantially associated with a lower incidence of stunting. The beneficial impact of owning a domestic animal on the decrease in stunting in children implies that apart from nutritional interventions in Bangladesh, enhancing livestock output could also aid in enhancing the nutritional condition of children. To maximize the effect, integrated programs, including agricultural production and WASH, are needed. A small effect size may be owing to the lack of dietary diversity, livestock health, and productivity data as well as the complexity of the relationship, requiring further studies [22].

Another study by Gelli et al [23] showed that the size of the chicken flock, poultry husbandry, and household hygiene standards were all correlated with the occurrence of chicken feces. Higher height-for-age *z* scores were linked to improved water sources and more visibly clean children. Lower weight-for-height *z* scores were linked to the presence of chicken feces; no correlations were detected with the height-for-age *z* scores. They also concluded that increasing poultry production in environments where children and poultry coexist may raise nutrition-related risks for young children, and more livestock-focused WASH programs may act as a significant barrier to stopping the spread of pathogens from livestock to humans [23].

A systematic review by Muema et al [24] suggested that depending on the type of intervention and length of program/intervention implementation, different livestock interventions have different effects on stunting, wasting, and underweight. Hence, due to the heterogeneity in reporting metrics, they were unable to compute the pool estimates. They further suggested that more randomized controlled trials with uniform and comparable reporting criteria are therefore required to strengthen the body of information regarding the effects of nutrition-sensitive livestock interventions on child development outcomes because the quality of the available data is low. They concluded that improving dietary diversity was a result of nutrition-sensitive livestock interventions, which increased the consumption of animal-source food [24].

Mosites et al [25] incorporated monthly anthropometric measurements for children younger than 5 years into an ongoing linked human and animal surveillance cohort in Western Kenya. Using linear mixed models, they tested whether baseline household livestock ownership was related to baseline child height-for-age or prospective growth rate. This study did not find a relationship between owning higher numbers of household livestock and child baseline anthropometric measures or subsequent child linear growth outcomes. However, there was an indication that livestock disease was associated with some measures of diminished growth [25].

### Limitations

Potential limitations that can impact the validity and reliability of the findings are as follows. Selection bias, the method of selecting cases and controls, may not accurately reflect the general population, leading to possible bias in the findings. Recall bias can be another limitation in this study, as this study will rely on the informant’s recollection of livestock ownership and child health history. Failure to consider the confounding factors can misrepresent the true association, adding to the limitations. Inaccuracies in anthropometry measurements of children can affect the study findings. Our findings will be from a specific region in central rural India, which may not be generalized to other regions with different cultural, environmental, and economic settings.

### Conclusions

There is a lack of studies in Maharashtra, India, which examine the relationship between livestock ownership and stunting in children younger than 5 years. Considering existing research gaps, this study aims to assess the relationship between stunting among children younger than 5 years in central rural India and livestock ownership by using the TLU index. It will determine the prevalence of the current stunting, wasting, and underweight among Indian children younger than 5 years. This study will offer insight into the correlations between stunting, wasting, and livestock ownership. This association will guide policymakers and program designers about the potential strategies to reduce stunting, such as promoting livestock ownership and ensuring that it provides nutritional benefits for children. This will be crucial in developing new public health policies, integrated programs, and nutritional interventions. It will also offer insights into how household resources (like livestock) are allocated and their impact on child nutrition and health, contributing to broader discussions about rural livelihoods and child development, particularly in a resource-constrained rural settings where understanding the determinants of child health is critical for developing effective interventions. It will help to generate hypotheses about the pathways through which livestock ownership might influence stunting, which can then be tested in future research such as cohort or interventional studies.

## Appendix

Multimedia Appendix 1 Study questionnaire.

## Abbreviations

NFHS-5: National Family Health Survey
TLU: Tropical Livestock Unit
WASH: Water, Sanitation, and Hygiene
WHO: World Health Organization
