# Supplementary material for: Association Between Household Livestock Ownership and Stunting in Children Younger Than 5 Years in Central Rural India: Protocol for a Case-Control Study
Source: JMIR Res Protoc. 2025 Sep 11;14:e66576. doi: 10.2196/66576 (PMC12464499; doi:10.2196/66576)
Supplement: Multimedia Appendix 1 [file resprot_v14i1e66576_app1.docx]

**QUESTIONNAIRE**

**Part A: Socio-demographic profile of the child, mother, and household.**

| **Socio-Demographic Information** | | | |
| --- | --- | --- | --- |
| **Sr.no.** | **Questions** | **Answers** | **Comments** |
| 1) | Date of Survey |  |  |
| 2) | Name of the Informant |  |  |
| 3) | Age of the informant |  |  |
| 4) | Relation of the informant with the child | 1. Mother  2. Father  3. Grandmother  4. Grandfather  5. Other |  |
| 5) | Mobile number |  |  |
| 6) | Adress |  |  |
| 7) | Name of the child |  |  |
| 8) | Gender | 1. Female 2. Male   3. Other |  |
| 9) | Date of Birth |  |  |
| 10) | Age of the child (In completed months) |  |  |
| 11) | Religion | 1. Hindu 2. Islam  3. Christian 4. Other |  |
| 12) | Caste | 1. General 2. Scheduled Caste (SC) 3. Scheduled Tribe (ST) 4. Other Backward Caste (OBC) 5. Special Backward Class (SBC) 6. Other |  |
| 13) | Education of Mother | 1. Illiterate 2. Didn’t go to school but can read and write 3. Primary - Standard 1^st^ to 4^th^ 4. Secondary - Standard 5^th^ to 10^th^ 5. Higher Secondary - Standard 11^th^ to 12^th^ 6. Diploma/Certificate course 7. Bachelor's or Higher education |  |
| 14) | Education of Father | 1. Illiterate  2. Didn’t go to school but can read and write   1. Primary - Standard 1^st^ to 4^th^ 2. Secondary - Standard 5^th^ to 10^th^ 3. Higher Secondary - Standard 11^th^ to 12^th^ 4. Diploma/Certificate course 5. Bachelors or Higher education |  |
| 15) | Employment status of the father | 1. Employed  2. Unemployed |  |
| 16) | If employed, then the occupation | 1. Farmer  2. Wage laborer  3. Skilled worker  4. Petty Trader (shopkeeper)  5. Self-employed  6. Service – Government  7. Service Private  8. Homemaker  9.Animal Husbandry  10. Others |  |
| 17) | The employment status of the mother | 1. Employed  2. Unemployed |  |
| 18) | If employed, then the occupation | 1. Farmer  2. Wage laborer  3. Skilled worker  4. Petty Trader (shopkeeper)  5. Self-employed  6. Service – Government  7. Service Private  8. Homemaker  9.Animal Husbandry  10. Others |  |
| 19) | Total monthly household income (in Rupees) |  |  |
| 20) | Type of family | 1. Nuclear  2. Joint family 3. Three-generation family |  |
| 21) | Total number of household members (Including self) | 1. 1 2. 2 3. 3 4. 4 5. 5 6. 6 7. 7   More than 7 |  |
| 22) | Housing Structure | 1. Hut 2. Semi Pucca House 3. Pucca House 4. Bungalow |  |
| 23) | Homeownership | 1. Rented  2. Owned |  |
| 24) | Do you have a Ration Card? | 1. Yes   0. No |  |
| 25) | If yes which color Ration | 1. Yellow (BPL)  2. Saffron (AI 15 K-1L)  3. White (AI 1 L >) |  |
| 26) | Total Number of rooms in the house | 1. 1 2. 2 3. 3 4. 4 5. 5 6. More than 5 |  |
| 27) | Is the Kitchen separate? | 1. yes  0. No |  |
| 28) | From where do you get your drinking water? | 1. Tap in the House 2. Common Tap 3. Hand pump / Bore well 4. Well 5. Pond 6. Others: (specify):_____________ |  |
| 29) | What type of cooking fuel do you use? | 1. LPG/Gas 2. Kerosene 3. Firewood 4. Gobar gas/biofuels 5. Others: Specify: |  |
| 30) | What toilet arrangements do you have? | 1. Private (in your own house 2. Common (shared by others) 3. Open defecation 4. Others: Specify: |  |
| 31) | Assets  *Multiple answers are allowed | 01. Radio  02. Television  03. Telephone  04. Motorbike or scooter  05. Car  06. Tractor  07. Land  08. Fridge  09. Air cooler  10. Geyzer  11. Refrigerator  12.Others___________________ |  |
| 32) | Distance (in km) to the nearest health center. |  |  |

**PART B) PERSONAL HISTORY OF THE CHILD**

| **Sr. no.** | **Questions** | **Answers** | **Comments** |
| --- | --- | --- | --- |
| 1) | Place of birth | 1. Institutional  2. Home |  |
| 2) | Type of delivery | 1. Normal Vaginal delivery 2. Assisted vaginal delivery 3. LS C-Section |  |
| 3) | Gestation age of the baby | 1. Preterm 2. Term 3. Post-term |  |
| 4) | Weight of the baby at birth (in grams) |  |  |
| 5) | Was exclusive breastfeeding done for 6 months | 1. Yes 2. No |  |
| 6) | Is the child fully immunized to date | 1. Yes  0.No |  |
| 7) | Diet Type | 1. Veg 2. Mixed 3. Non-veg |  |
| 8) | Type of Animal source food (ASF) consumed | 1. Milk 2. Egg 3. Meat 4. Panner 5. Other |  |
| 9) | Number of times ASF intake in a week | 1. 1 2. 2 3. 3 4. 4 5. 5 6. 6   7. 7 |  |

**TWO-WEEK RECALL FOR CHILD-ILLNESS.**

| **Sr.no.** | **Question** | **Answer** | **Comments** |
| --- | --- | --- | --- |
| 1. | Has [Child's Name] had diarrhea in the last 2 weeks? | 1. Yes  2. No  3. Don’t know |  |
| 2. | If yes: a. Number of days with diarrhea: |  |  |
|  | Was blood in the stool? | 1. Yes  0.No |  |
|  | Was treatment sought? | 1. Yes  0.No |  |
| 3. | Has [Child's Name] had a fever in the last 2 weeks? | 1. Yes  2. No  3. Don’t know |  |
|  | If yes: a. Number of days with fever: |  |  |
|  | b. Was treatment sought? | 1. Yes  0.No |  |
| 4. | Has [Child's Name] had a cough or difficult/fast breathing in the last 2 weeks? | 1. Yes  0.No |  |
|  | If yes  a.Was treatment sought? | 1. Yes  0.No |  |

**Section c) Anthropometric measurements.**

| 1) | Length height (in cms) |  |  |
| --- | --- | --- | --- |
| 2) | Weight (in kg) |  |  |
| 3) | MUAC (in cms) |  |  |
| 4) | Head circumference (in cms) |  |  |

**Section D) Livestock survey**

| **Sr.no** | **Question** | **Answers** | **Comments** |
| --- | --- | --- | --- |
| 1. | Do you own any livestock? | 1. yes  0. no |  |
| **Section 1: General demographics information** | | | |
| 1. | Total number of Cows |  |  |
| 2. | Total number of buffalos |  |  |
| 3. | Total number of goats |  |  |
| 4. | Total number of poultry (cocks and hens) |  |  |
| 5. | Total number of pigs |  |  |
| 6. | Total number of sheep |  |  |
| 7. | Total number of livestock |  |  |
| 8. | Total TLU |  |  |
| 9. | Distance from the cattle shed to the house (In feet) |  |  |
| 10. | Approximately how many minutes a day does your child play/spend around these livestock? | 1.0 minutes  2. 30 minutes  3. 60 minutes  4. 90 minutes  5.120 minutes  6. more than 120 minutes |  |
| 11. | Where do you keep the chickens overnight? | 1. In the house. 2. Outside the house. |  |
| 12. | If in the house | 1. Caged 2. Free |  |
| **Section 2: Cattle shed** | | | |
| **1.** | Is there any designated space for keeping and rearing cattle? | 1. Yes  0. No |  |
| **2.** | Where do you generally keep your cattle | 1. In the compound of the house  2. Off compound  3. On road  4. Away from house  5. Others__________ |  |
| **3.** | If yes, what kind of place? | 1. Well-structured tie-stall (Tabela)  2. Semi-structured tie-stall  3. Unstructured tie-stall  4. No tie-stall |  |
| **4.** | What kind of roof over a cow shed? | 1. Cement roof  2. Tin shed  3. Plastic shed  4. Straw shed  5. No roof  6. Others__________ |  |
| **5.** | What kind of walls around cow shed? | 1. Cement  2. Tin  3. Plastic  4. Mud  5. No walls  6. Others__________ |  |
| **6.** | What kind of floor in a cow shed? | 1. Cement  2. Soil  3. Cow dung  4. Others__________ |  |
| **7.** | Where do you dispose the cow dung? | 1. In compound of house  2. Off compound  3. On road  4. Away from house  5. Others__________ |  |

**Section E) Environmental health survey**

| **Sr.no.** | **Question** | **Answer** | **Comments** |
| --- | --- | --- | --- |
| **Section 1: Water** | | | |
| 1. | What is the main source of drinking water for members of your household? | 1. Piped into dwelling  2. Public Tap  3. Tap at Neighbor  4. Own Standpipe  5. Public Standpipe  6. Neighbor’s Standpipe  7. Tanker Truck  8. Bottled Water  9. Other (Specify) ____________________ |  |
| 2. | Generally, water from water sources is free from turbidity | 1. Yes 2. No |  |
| 3. | Storage of water | | |
|  | For Drinking water | Water for Basic HH activities | Water for Animals |
|  | 1. Direct use from tap 2. Pipe 3. Matka 4. Bucket and mug 5. Another utensil 6. Water tank 7. Drum (add)   Others | 1. Direct use from tap 2. Pipe 3. Matka 4. Bucket and mug 5. Other utensil (remove option from this table) 6. Water tank 7. Drum (add)   Others | 1. Direct use from tap 2. Pipe 3. Matka (remove option from this table) 4. Bucket 5. Other utensil (remove option from this table) 6. Water tank 7. Drum (add)   Others |
| **Section 2: Sanitation** | | | |
| 1. | What kind of toilet facility do members of your household usually use? | 1. Open defecation 2. Public Toilet 3. Public Pay and Use Toilet 4. Family’s private toilet |  |
| 2. | Currently, Water is available for flushing | 1. Yes 2. No |  |
| 3. | At present, is the child using the toilet at home? | 1. Yes 2. No |  |
| 4. | If no, then where does your child defecate? | 1. Just in front of the house 2. Just out of the compound 3. Away from the compound 4. Other______________ |  |
| 5. | Does the child wash hands after defecation? | 1. Yes   0.No |  |
| 6. | If yes, hand wash techniques | 1. With liquid soap 2. With soap 3. Without soap only with water 4. If any other ___ |  |
| **Section 3: Personal Hygiene** | | | |
| 1. | How many times a day do you wash your hands with soap | 1. 0 time 2. 1 - 3 time 3. 4 - 6 time 4. 7 - 10 time 5. >10 time |  |
| 2. | How many times a day do you wash your hands without soap | 1. 0 time 2. 1 - 3 time 3. 4 - 6 time 4. 7 - 10 time   >10 time |  |
| 3. | How many times a day does the child wash their hands with soap | 1. 0 time 2. 1 - 3 time 3. 4 - 6 time 4. 7 - 10 time   >10 time |  |
| 4. | How many times a day does the child wash their hands without soap | 1. 0 time 2. 1 - 3 time 3. 4 - 6 time 4. 7 - 10 time 5. >10 time |  |
| **Section 4: Domestic Hygiene** | | | |
| 1. **Household cleaning details** | | | |
| 1. | Currently how many times a day do you mop house flooring? | 1.0  2.1  3.2  4.3  5.4  6. more than 4 |  |
| 2. | At present Do you use any chemicals to clean/shine floors | 1. Yes   0.No |  |
| 3. | How many times a day do you clean kitchen surfaces? | 1.0  2.1  3.2  4.3  5.4  6. more than 4 |  |
| 4. | Do you generally clean floors before you sit to eat? | 1. Yes   0.No |  |
| 5. | If yes, how do you clean it? | 1. By using broom 2. By using mop   Both |  |
| 6. | How many times a week do you clean compound | 1.0  2.1  3.2  4.3  5.4  6.5  7.6  8.7  9. more than 7 |  |
| 7. | How many times a month do you clean the walls of the house | Inside house _______  1.0  2.1  3.2  4.3  5.4  6.5  7. more than 5 |  |
| 8. | Do you dump garbage surrounding the house | 1. Yes   0.No |  |
| 9. | Is there any cow dung dump surrounding the house | 1. Yes   0.No |  |
| 1. **Cattle shed cleanliness** | | | |
| 1. | Do you clean the cattle shed daily | 1. Yes   0.No |  |
| 2. | How many times a week do you clean the shed? |  |  |
| 3. | Do you use any chemicals to clean the shed? | 1. Yes   0.No |  |

**Section G) Knowledge, Attitude and Practice.**

| **Sr.no** | **Question** | **Answer** | | | | **Comments** |
| --- | --- | --- | --- | --- | --- | --- |
| 1. | Have you ever heard that animals you handle can be a cause of disease? | 1. Yes   2.No | | | |  |
| 2. | If yes which kind of diseases name have you heard? |  | | | |  |
| 3. | What are the symptoms of these diseases? |  | | | |  |
| 4. | How it spreads from animal to human | 1. Milk 2. Meat 3. Air 4. Feed 5. Contact to infected animal 6. Soil 7. Contact to contaminated excreta of animals   Other______________ | | | |  |
| 5. | Which kind of practices do you do? | 1. Sleeping with animal/in animal shield 2. Consumption of raw milk, eggs & meat 3. History of animal abortion at the farm 4. Disposed off aborted fetus with naked hands 5. Disposed off placenta without gloves 6. Intrauterine medication after abortion 7. Apply milk on cracked lip   8.Others______________________ | | | |  |
| 6. | Are you aware about any prevention strategy of zoonotic diseases? | 1. Yes 2. No | | | |  |
| 7. | What kind of preventive measure do you take? | 1. Testing of new animal for brucellosis and TB 2. Regular Veterinary check up 3. Hand Wash 4. Wearing gloves 5. Use of mask 6. Boot wearing 7. Avoid contact with naked hands 8. Disposal of animal carcass 9. Boiling milk before drink 10. Avoid raw meat eating 11. Other______________________________________ | | | |  |
|  |  | Self | | | Family members |  |
| 8. | Have you ever suffered from any of the following zoonotic diseases | - Rabies - Brucellosis - Tuberculosis - Anthrax - Bird flue - CCHF - Leptospirosis - Q-fever   Others_________________ | | - Rabies - Brucellosis - Tuberculosis - Anthrax - Bird flue - CCHF - Leptospirosis - Q-fever   Others___________ | |  |
| 9. | Have you ever experienced the following | - Respiratory Infection - Digestive Disturbance (Diarrhea + vomiting) - Dermatological problem - Fever - Body pain - Headache - Joint pain   Others | - Respiratory Infection - Digestive Disturbance (Diarrhea + vomiting) - Dermatological problem - Fever - Body pain - Headache - Joint pain   Others_______________ | | |  |

**Section F) Spot check.**

| **Sr. No.** | **Question** | **Answers** | **Observation** |
| --- | --- | --- | --- |
| **Section 1: Water** | | | |
| 1 | General water source | *Write source of water  _________________________ |  |
| 2 | Water is available in the house from source | 1. Yes   No |  |
| 3 | Water source visibly clean | 1. Yes   No |  |
| 4 | Water storage container visibly clean | 1. Yes   0.No |  |
| 5 | Water storage container covered | 1. Yes   0.No |  |
| 6 | Adequate withdrawal method | 1. Yes   0.No |  |
| **Section 2: Sanitation** | | | |
| 1. | Type of sanitation | 1. Improved   Unimproved | Type of toilet   1. Indian   Western |
| 2. | Open deification | 1. Yes   0.No |  |
| 3. | Water available for flushing | 1. Yes   0.No |  |
| 4. | Water available for handwashing after using sanitation facility | 1. Yes   0.No |  |
| 5. | Visible fecal contamination | 1. Yes   0.No |  |
| **Section 3: Personal Hygiene** | | | |
| 1. | Visible sign of dirt in finger nails | 1. Yes   0.No |  |
| 2. | Black or red teeth | 1. Yes   0.No |  |
| 3. | Dirty clothes | 1. Yes   0.No |  |
| **Section 4: Domestic Hygiene** | | | |
| 1. | Significant numbers of flies | 1. Yes   0.No |  |
| 2. | Household floors are visibly clean and free from dust | 1. Yes   0.No |  |
| 3. | Household walls are visibly clean and free from dust | 1. Yes   0.No |  |
| 4. | Household walls and floors are free from moisture | 1. Yes   0.No |  |
| 5. | There is enough ventilation in the house | 1. Yes   0.No |  |
| **Section 5: Livestock Hygiene** | | | |
| 1. | The cattle shed is visibly clean | 1. Yes   0.No  2.NA |  |
| 2. | Drinking water for animals is visibly clean | 1. Yes 2. No   NA |  |
| 3. | The surrounding compound is visibly clean | 1. Yes   0.No |  |
| 4. | Surrounding compound is free from cow dung dump | 1. Yes   0.No |  |
| 5. | Cattle surrounding non-livestock keeper's house | 1. Yes   0.No  2.NA |  |
